# Regulation and Rate Enhancement during Transcription-Coupled DNA Repair

**DOI:** 10.1016/j.molcel.2010.11.012

**Published:** 2010-12-10

**Authors:** Laura Manelyte, Young-In T. Kim, Abigail J. Smith, Rachel M. Smith, Nigel J. Savery

**Affiliations:** 1 DNA-Protein Interactions Unit, School of Biochemistry, University of Bristol, Bristol BS8 1TD, UK

## Abstract

Transcription-coupled DNA repair (TCR) is a subpathway of nucleotide excision repair (NER) that is triggered when RNA polymerase is stalled by DNA damage. Lesions targeted by TCR are repaired more quickly than lesions repaired by the transcription-independent “global” NER pathway, but the mechanism underlying this rate enhancement is not understood. Damage recognition during bacterial NER depends upon UvrA, which binds to the damage and loads UvrB onto the DNA. Bacterial TCR additionally requires the Mfd protein, a DNA translocase that removes the stalled transcription complexes. We have determined the properties of Mfd, UvrA, and UvrB that are required for the elevated rate of repair observed during TCR. We show that TCR and global NER differ in their requirements for damage recognition by UvrA, indicating that Mfd acts at the very earliest stage of the repair process and extending the functional similarities between TCR in bacteria and eukaryotes.

## Highlights

► Bacterial TCR bypasses the need for damage recognition by UvrA ► The UvrB homology module of Mfd is responsible for strand-specific repair ► The insertion domain of *E. coli* UvrA is involved in damage recognition ► Interdomain contacts regulate the activity of Mfd

## Introduction

Transcription-coupled DNA repair (TCR) is a subpathway of nucleotide excision repair (NER) that targets DNA lesions in the template strand of active genes and plays an important role in maintaining genome integrity (Hanawalt and Spivak, 2008). TCR is initiated when RNA polymerase (RNAP) stalls at a lesion and typically results in damage being repaired more quickly than it would be in nontranscribed regions that are repaired by the global NER pathway. All organisms in which TCR has been detected express a transcription-repair coupling factor (reviewed in Hanawalt and Spivak, 2008; Svejstrup, 2002). These proteins have been characterized in humans (CSB), yeast (Rad26), and bacteria (Mfd) and are all ATP-dependent enzymes that interact with the transcription apparatus and with components of the NER apparatus.

NER has a broad substrate specificity, and the lesions that it targets are detected via multistep processes (Shuck et al., 2008; Truglio et al., 2006a). Damage recognition during global NER in bacteria is performed by a complex of UvrA and UvrB proteins. The stoichiometry of this complex is uncertain, with UvrA_2_UvrB_2_ tetramers being detected in some studies (Kad et al., 2010; Malta et al., 2007; Verhoeven et al., 2002) and UvrA_2_UvrB trimers in others (Orren and Sancar, 1989; Pakotiprapha et al., 2009). Both UvrA and UvrB participate in damage detection. UvrA is responsible for initial recognition and loads UvrB onto DNA, possibly by partially unwinding the DNA close to the lesion. UvrB inserts a β-hairpin between the two strands, clamping one against an adjacent protein domain (Truglio et al., 2006b). Limited ATP-dependent DNA translocation by UvrB may be required for final lesion verification. At this stage, UvrA dissociates from the complex and is replaced by UvrC, which cuts the damaged strand on either side of the lesion. The short oligonucleotide that results is displaced by UvrD helicase, and the gap in the damaged strand is filled by DNA polymerase I and DNA ligase.

During TCR, the inability of RNAP to transcribe a noncoding lesion in the template strand causes it to stall, thus denying repair proteins access to the damage (Selby and Sancar, 1990). Mfd is recruited to the stalled transcription complex via a specific interaction with RNAP and removes RNAP from the DNA (thus overcoming the inhibitory effect that RNAP stalled at a lesion has on repair) (Park et al., 2002; Selby and Sancar, 1993; Smith and Savery, 2005). As TCR is faster than global NER, Mfd and/or RNAP must also affect the rate at which the Uvr proteins undertake repair. The mechanism by which this rate enhancement occurs is not known, but Mfd can interact with UvrA, and this interaction is presumed to be important for TCR (Selby and Sancar, 1993, 1995a). It has been suggested that this interaction recruits UvrA to damaged DNA or that it promotes dissociation of UvrA from a UvrA:UvrB:DNA preincision complex (Selby and Sancar, 1993, 1994). Models for strand-specific repair mechanisms that are independent of Mfd-UvrA interactions have also been proposed (Kunala and Brash, 1995; Patel et al., 2004).

**Figure 1:**
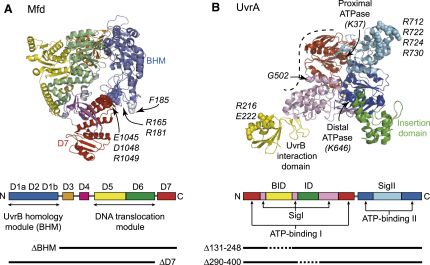
Mfd and UvrA Proteins (A) Structure of *E. coli* Mfd (PDB ID 2EYQ) (Deaconescu et al., 2006). Residues mutated in this study are shown in space-filling representation, and the truncated proteins Mfd ΔBHM and Mfd ΔD7 are shown as black bars. (B) Structure of *B. stearothermophilus* UvrA (PDB ID 2R6F) (Pakotiprapha et al., 2008). A single monomer is shown, and the dimerization interface is indicated by a dotted line. SigI and SigII domains contain ABC ATPase signature motifs. Residues equivalent to those mutated in this study are shown in space-filling representation (labels show *E. coli* residues and numbering). The truncated proteins UvrA Δ131-248 and UvrA Δ290-400 are shown as black bars, with dotted lines indicating regions missing from the constructs.

Mfd is an eight-domain monomeric protein (Deaconescu et al., 2006) (Figure 1A). Domain 4 is the RNAP-binding domain, and domains 5 and 6 comprise a helicase superfamily 2 DNA translocation module. Domains 1a, 2, and 1b are structurally homologous to 3 of the 5 domains of UvrB and are termed the UvrB homology module (BHM). In UvrB, these domains are involved in interaction with UvrA and UvrD, in damage recognition, and in ATP hydrolysis (Truglio et al., 2006a). The motifs responsible for damage recognition and ATP hydrolysis are absent from the BHM of Mfd, and the sequence similarity between the two proteins is greatest in domain 2 (D2) (Deaconescu et al., 2006; Selby and Sancar, 1993). Although little is known about the Mfd:UvrA interaction, the UvrB:UvrA interaction has been characterized by mutagenic studies (Truglio et al., 2004) and by cocrystallization of UvrB D2 in complex with its partner domain from UvrA (Pakotiprapha et al., 2009). By analogy to UvrB, the surface of Mfd D2 that is likely to interact with UvrA can be deduced (Deaconescu et al., 2006; Pakotiprapha et al., 2009). In the crystal structure of apo-Mfd, this surface is largely buried in an intramolecular contact with domain 7 (D7), suggesting that interdomain rearrangements would be necessary to allow the crystallized form of the protein to interact with UvrA (Deaconescu et al., 2006). Interdomain rearrangements involving D7 are also thought to be necessary for activating the DNA translocation activity of Mfd when it binds to RNAP, as deletion of either D7 or domains 1–3 relieves the autoinhibition of motor activity that is observed in the isolated full-length protein (Murphy et al., 2009; Smith et al., 2007).

The mechanism of RNAP displacement by Mfd is becoming clearer. The ATP-dependent DNA translocase activity of Mfd is activated when the protein binds to a stalled transcription complex (Smith et al., 2007). This pushes RNAP forward and destabilizes the interactions that hold the transcription complex in place (Park et al., 2002). In contrast, the mechanism by which the rate of DNA repair is enhanced during TCR remains poorly understood. In this work, we have investigated the determinants of TCR in Mfd, UvrA, and UvrB using mutants in which individual functions of each protein were specifically impaired. We show that the BHM of Mfd is essential for the enhancement of repair rate but that the regulation of UvrA binding and DNA translocation by D7 is not. We also show that the damage specificity of UvrA is at least partially redundant during TCR but that TCR retains the need for damage recognition by UvrB.

## Results

### The UvrA Interaction Surface Is Involved in the Autoregulation of Mfd

The interaction between UvrA and the BHM of Mfd is presumed to be essential for the elevated rate of repair that is observed during TCR, but this hypothesis has not been tested experimentally. To address this issue, we constructed Mfd derivatives in which the putative UvrA interaction surface was either disrupted by substitutions or removed entirely by deletion of the BHM. Mfd R165A R181A F185A (Mfd D2AAA) carries alanine substitutions of three residues on the surface of Mfd D2 that, on the basis of homology with UvrB, have been highlighted in previous studies as potential UvrA-interacting residues (Assenmacher et al., 2006; Deaconescu et al., 2006). In the structure of Mfd, residues R165 and R181 are buried in the interface of D2 and D7, whereas F185 is exposed (Figure 1A). These three residues are conserved between Mfd and UvrB in *E. coli* and are all highly conserved among Mfd proteins from different species. Mfd ΔBHM is an N-terminally truncated derivative of *E. coli* Mfd that lacks domains 1a, 1b, and 2 (Figure 1A).

**Figure 2:**
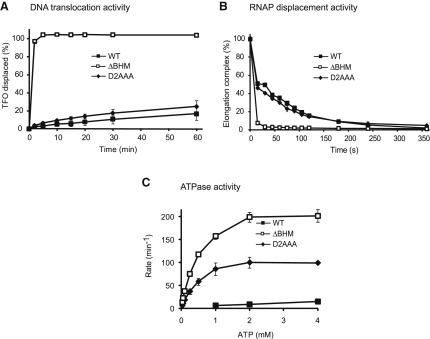
Biochemical Analysis of Mfd Derivatives Containing Mutations within the UvrB Homology Domain (A) DNA translocation activity. Displacement of a triplex-forming oligonucleotide (TFO) from supercoiled pSRTB1 plasmid was monitored by EMSA. The graph shows the percentage of TFO displaced at each time point, normalized for the amount of triplex present at t = 0. Data are the mean of at least three independent experiments and are shown with standard deviation. (B) RNAP displacement activity. Transcription complexes were stalled by nucleotide starvation on a PCR product carrying the T7A1 promoter, and displacement by Mfd was monitored by EMSA. The graph shows the percentage of the transcription elongation complex present at t = 0 that was displaced at each time point. Data are the mean of at least three independent experiments and are shown with standard deviation. (C) ATPase activity. Rates were measured at 37°C using an NADH-coupled assay. Data are the mean of at least three independent experiments and are shown with standard deviation.

We purified Mfd D2AAA and Mfd ΔBHM and examined their ATPase, DNA translocation, and RNAP displacement activities (Figure 2). Mfd ΔBHM had deregulated ATPase and DNA translocase activities and displaced stalled transcription complexes from DNA more quickly than WT Mfd did. It thus behaved like previously characterized Mfd derivatives in which the autoinhibition of the protein was disrupted by N- or C-terminal truncations (Murphy et al., 2009; Smith et al., 2007). Mfd D2AAA was unable to translocate DNA in the absence of RNAP and displaced RNAP from DNA at a rate similar to the WT protein. However, the ATPase activity of Mfd D2AAA was considerably higher than WT, and although its maximal activity was lower than that of Mfd ΔBHM, it was comparable to that of Mfd ΔD7 (∼110 min^−1^) (Smith et al., 2007). The properties of Mfd D2AAA suggest that the substitutions within the UvrA interaction surface may affect the interaction of D2 with D7 and thus partially relieve the autoinhibition of the translocase domains. To investigate this possibility further, we determined the effect of disrupting the highly conserved cluster of residues within D7 that interact with D2 (Deaconescu et al., 2006). We constructed a derivative of Mfd in which residues E1045, D1048, and R1049 were all substituted with alanine (Figure 1A) and examined the activities of the purified protein (Figure S1). We found that Mfd E1045A D1048A R1049A retained the ability to displace stalled RNAP and exhibited the elevated ATPase rate and robust RNAP-independent translocase activity that is a signature of derepressed Mfd derivatives. Taken together, our results indicate that the derepression of Mfd ATPase/translocase activity, which has previously been reported for truncated Mfd derivatives lacking one or more domains, can be effected in the full-length protein by amino acid substitutions in either of the surfaces that constitute the D2:D7 interaction.

### The UvrB Homology Module of Mfd, but Not Domain 7, Is Essential for TCR In Vitro

**Figure 3:**
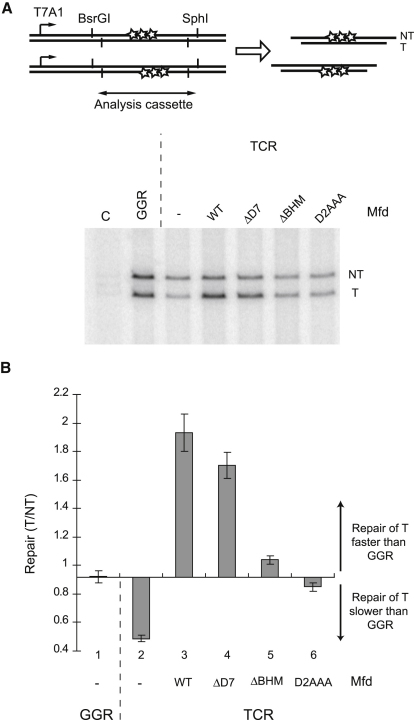
Effect of Mfd Derivatives on TCR In Vitro (A) TCR was reconstituted in vitro using purified proteins and a UV-irradiated plasmid substrate. The plasmid contained a “reporter cassette” flanked by BsrGI and SphI sites, downstream of the T7A1 promoter. Repair was monitored by incorporation of [α-^32^P]dATP into repair patches, and cleavage by BsrGI and SphI to produce nontemplate strand (NT) and template strand (T) fragments. The figure shows the analysis of the products by denaturing gel electrophoresis. TCR reactions contained RNAP, UvrA, UvrB, UvrC, UvrD, DNA ligase, DNA polymerase, and where indicated, Mfd or an Mfd derivative. Global NER reactions (GGR) were performed as for TCR reactions, except that RNAP and Mfd were omitted. The control reaction in lane C was performed as for the GGR reaction, except that UvrA, UvrB, UvrC, and UvrD were omitted. (B) Quantification of strand bias. The figure shows the ratio of incorporation of radioactive label into the T and NT strands under the conditions indicated. Data are the mean of at least three independent experiments and are shown with standard deviation.

To determine which aspects of Mfd function are required for strand-specific repair, we established a patch-synthesis assay to monitor repair of UV-induced lesions in vitro. RNAP, Mfd, repair proteins, and all necessary cofactors were incubated with UV-irradiated plasmid DNA, and repair was monitored by the incorporation of [α-^32^P]dATP into repair patches. The template (transcribed) and nontemplate strands of a “reporter cassette” downstream of the strong T7A1 promoter were distinguished by denaturing gel electrophoresis following asymmetric cleavage by restriction enzymes (Figure 3A). The nontemplate strand is repaired by the global NER pathway and is not a substrate for TCR (Selby and Sancar, 1993) (Figure S2A). The efficiency of repair of the template strand relative to the rate of global NER can thus be deduced by comparing the amount of radiolabel incorporated into the template and nontemplate strands.

In the absence of RNAP, repair patches formed in the two strands with approximately equal efficiency, as both strands are repaired by global NER (Figure 3B, lane 1). As expected from previous studies of TCR (Selby and Sancar, 1993), transcription in the absence of Mfd specifically inhibited repair of the template strand (Figure 3B, lane 2), and transcription in the presence of WT Mfd specifically enhanced repair of the template strand (Figure 3B, lane 3). The Mfd-dependent strand bias in our assay was sensitive to UvrA concentration (Figure S2B), as reported previously (Selby and Sancar, 1995b).

When Mfd ΔBHM or Mfd D2AAA was used in the assay, there was little strand bias in the synthesis of repair patches (Figure 3B, lanes 5 and 6), but Mfd ΔD7 catalyzed the preferential repair of the template strand almost as efficiently as WT Mfd did (Figure 3B, lane 4). We conclude that all three mutant proteins displaced transcription complexes stalled at lesions, thus preventing the inhibition of template strand repair, but that only Mfd ΔD7 retained the ability to enhance the repair of the template strand. The simplest interpretation of these results is that the interaction of D2 of Mfd with UvrA is essential for the mechanism by which repair is accelerated during TCR, and that substitution of conserved residues within the D2 surface or deletion of the BHM disrupts this interaction. The observation that Mfd ΔD7 can catalyze strand-specific repair indicates that autoregulation of UvrA binding and DNA translocation by Mfd is dispensable for the mechanism by which strand-biased repair is achieved. Further support for this proposal comes from the observation that the deregulated Mfd E1045A D1048A R1049A mutant can also catalyze strand-specific repair (Figure S1D).

### The UvrB Homology Module of Mfd, but Not Domain 7, Is Essential for TCR In Vivo

**Figure 4:**
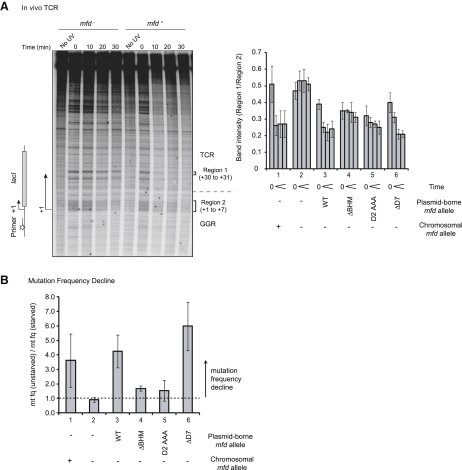
Effect of Mfd Derivatives In Vivo (A) Primer extension analysis of TCR in strains transformed with a plasmid carrying a *lacI* gene. Samples were taken before UV irradiation (No UV), immediately following irradiation (0), and at intervals after incubation in the dark at 37°C. Plasmid DNA isolated from each sample was analyzed using a primer that annealed to the template strand upstream of the *lacI* promoter. The gel image shows the primer extension products obtained from cultures of strain UNCNOMFD transformed with pET21 (*mfd^−^*) or pETMfd2 (*mfd^+^*). The bands reflect the location of lesions within the *lacI* template strand. The first transcribed base and direction of transcription are indicated by a bent arrow. A dotted line marks the transition between the region repaired by GGR and the region repaired by TCR at +15. Box brackets indicate the regions whose intensity was quantified. The chart shows the relative intensities of bands in regions 1 (+30/+31) and 2 (+1 to +7). The four bars in each group represent samples taken at 0, 10, 20, and 30 min postirradiation. Group 1: AB1157 transformed with pET21a. Group 2: UNCNOMFD transformed with pET21a. Groups 3–6: UNCNOMFD transformed with pETMfd2 derivatives carrying the *mfd* alleles indicated. Data are the mean of at least three independent experiments and are shown with standard deviation. (B) Mutation frequency decline. The frequency of reversion of UV-irradiated *argE3(Oc)* strains to Arg^+^ was determined in samples that had either been added to rich medium immediately after irradiation (mt fq [unstarved]) or that had been held in a medium lacking amino acids for 30 min prior to addition of rich medium (mt fq [starved]). A mt fq (unstarved) to mt fq (starved) ratio of greater than 1 is indicative of MFD. Bar 1: AB1157. Bar 2: UNCNOMFD transformed with pET21a. Bars 3–6: UNCNOMFD transformed with pETMfd2 derivatives carrying the *mfd* alleles indicated. Data are the mean of at least three independent experiments and are shown with standard deviation.

To determine whether the results obtained in the in vitro TCR assay reflect the situation within a cell, we used a primer extension assay to detect repair in vivo. UV-induced lesions can block DNA polymerase, and analysis of the primer extension products allows detection of lesions at single-nucleotide resolution (Wellinger and Thoma, 1996). Our experiments monitored the generation and repair of lesions within the template strand of a plasmid-borne *lacI* gene in *mfd^+^* and *mfd^−^* cells (Figure 4A). Because the primer annealed upstream of the promoter, the experiment allowed analysis of both the untranscribed promoter DNA and the transcribed region of the *lacI* gene. Mfd-dependent TCR does not occur in untranscribed regions or in the promoter-proximal regions that are transcribed prior to dissociation of the sigma subunit of RNAP (Selby and Sancar, 1995b). In these experiments, the DNA between the primer and approximately +15 thus acted as a control region in which repair was independent of Mfd, and the effect of Mfd and Mfd mutants could be assessed in the region downstream of approximately +15.

Comparison of the primer extension products obtained from samples taken before and immediately after irradiation revealed the presence of premature termination products unique to the irradiated DNA (Figure 4A). The pattern of the predominant damage-induced bands correlated with the location of pyrimidine dimers and previously published analysis of UV-induced lesions in the *lacI* gene (Chandrasekhar and Van Houten, 1994; Kunala and Brash, 1995). However, the primer extension assay detects any damage that stalls Taq DNA polymerase, and it is likely that not all of the lesions detected were pyrimidine dimers. The rate at which individual lesions were repaired (detected as the disappearance of the primer extension product terminating at that location) differed considerably. Some lesions within the transcribed region of the *lacI* gene were repaired much more rapidly in the presence of Mfd than in its absence. Other lesions showed little repair in either strain, even after 30 min. One region in which an Mfd-dependent effect was clear was a pair of lesions that produced bands at +30/+31. These correspond to a known mutation “hotspot” in cells lacking Mfd (Kunala and Brash, 1992; Oller et al., 1992) and were chosen as the test lesions for assessing the effects of Mfd mutants.

To quantify the effect of Mfd and Mfd derivatives on repair, and to avoid the effects of lane-to-lane variations, we calculated the ratio of the intensity of the +30/+31 bands to the intensity of a group of bands from +1 to +7 at each time point. Lesions from +1 to +7 of genes are not subject to TCR, and we reasoned that if the lesions at +30/+31 were repaired at an enhanced rate, the ratio would decrease over time, whereas if the lesions at +30/+31 were repaired by the same global NER pathway as the +1 to +7 region, the ratio would remain constant as repair progressed. In agreement with these predictions, the ratio remained constant throughout the course of the experiment in cells that lacked Mfd (Figure 4A, group 2). In strains that expressed WT Mfd from either a chromosomal or plasmid-borne allele, the ratio decreased within the first 10 min, reflecting preferential repair of the lesions at +31/32, and then remained constant for the remainder of the experiment (Figure 4A, groups 1 and 3).

To examine the effects of Mfd mutants on TCR in vivo, experiments were conducted using *mfd^−^* cells transformed with plasmids carrying the mutant *mfd* alleles. Preferential repair of the lesions at +30/+31 was observed in cells containing Mfd ΔD7 (Figure 4A, group 6), but not in cells containing Mfd ΔBHM or Mfd D2AAA (Figure 4A, groups 4 and 5). TCR occurred over a longer period with Mfd ΔD7 than with the WT protein, which may reflect the sequestration of UvrA by the unshielded D2 of Mfd ΔD7 (Deaconescu et al., 2006; Selby and Sancar, 1995a).

A phenotype linked to TCR in *E. coli* is mutation frequency decline (MFD), which is a reduction in the frequency of certain suppressor mutations when cells are transiently starved of amino acids after UV irradiation (Selby and Sancar, 1994). The Mfd protein is essential for this phenotype, which is thought to be due to enhanced repair of lesions within tRNA genes. We examined the effect of Mfd mutants on the MFD phenotype by determining the frequency with which Arg^+^ revertants arose in *argE3_(ochre)_* strains. As expected, in cells expressing WT Mfd from either a chromosomal or plasmid-borne allele, the mutation frequency was lower in cells that were starved for 30 min after UV irradiation than in cells that were not starved (Figure 4B, lanes 1 and 3). Cells that lacked Mfd did not exhibit MFD in response to starvation (Figure 4B, lane 2). Mfd ΔD7 was able to restore the MFD phenotype to *mfd^−^* cells, but cells expressing Mfd ΔBHM or Mfd D2AAA showed little or no starvation-dependent MFD (Figure 4B, lanes 4–6).

The results of the in vivo TCR and MFD assays confirm those of the in vitro TCR assays, i.e., the BHM (and specifically the UvrA interaction surface of D2) is essential for the enhanced repair of lesions within the template strand and for MFD, but the regulatory role of D7 is dispensable for these processes.

### Substitutions within Mfd D2 Disrupt the Mfd:UvrA Interaction

**Figure 5:**
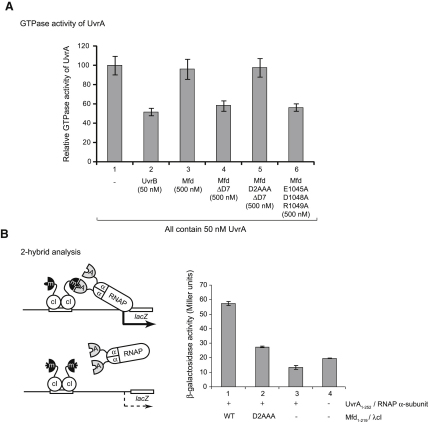
Interactions with UvrA (A) Effect of UvrB and Mfd on the GTPase activity of UvrA. Rates were measured at 37°C using an NADH-coupled assay and 2 mM GTP. Data are the mean of at least three independent experiments and are shown with standard deviation. (B) Bacterial two-hybrid analysis: A fragment of UvrA containing the UvrB interaction domain was fused to the α subunit of RNAP, and a fragment of Mfd containing D2 was fused to the λcI protein. Interaction between the fusion proteins recruits RNAP to a *lacZ* reporter construct, and β-galactosidase activity reflects the strength of the interaction. The chart shows the specific β-galactosidase activity measured in KS1 cells transformed with pRA02 and pRA03 derivatives encoding the indicated combinations of fusion proteins. “−” indicates expression of λcI or α subunit without additional fused domains. Data are the mean of at least three independent experiments and are shown with standard deviation.

When UvrA and UvrB interact, nucleotide hydrolysis by UvrA is inhibited and nucleotide hydrolysis by UvrB is increased (Caron and Grossman, 1988). The inhibition of UvrA by UvrB can be observed if GTP hydrolysis is monitored, because UvrB does not hydrolyze GTP (Caron and Grossman, 1988) (Figure 5A, compare bars 1 and 2). We found that domains 1a, 2, and 1b of UvrB (which are conserved in Mfd) are sufficient to regulate UvrA (Figure S3A), and so we investigated whether Mfd regulates the NTPase activity of UvrA. Mfd has no detectable GTPase activity (data not shown), and even at high concentration, WT Mfd had no effect on the GTPase activity of UvrA (Figure 5A, bar 3). Because the UvrA interaction surface is obscured in full-length Mfd by its interaction with D7, we also examined the effect of Mfd ΔD7 (in which D7 is deleted) and Mfd E1045A D1048A R1049A (in which the D2:D7 interface is disrupted by substitutions within D7). Both of these mutant proteins inhibited the GTPase activity of UvrA (Figure 5A, bars 4 and 6, and Figure S3B), which supports the hypothesis that in WT Mfd, the UvrA interaction surface is masked by its interaction with D7, and that a conformational change enables the proteins to interact.

The ability of Mfd ΔD7 to repress UvrA GTPase activity was abolished when the truncated Mfd contained the D2AAA substitutions (Figure 5A, compare bars 4 and 5). The D2AAA substitutions also disrupted Mfd:UvrA interactions in a bacterial two-hybrid system in which the strength of the interaction between protein fragments is reflected by β-galactosidase activity from a *lacZ* reporter construct (Figure 5B, compare bars 1 and 2). These results indicate that the D2AAA substitutions disrupt the Mfd:UvrA interaction and support the proposition that the UvrA interaction surface of Mfd is similar to that defined in UvrB.

### Mutations in UvrA Have a Differential Effect on TCR and Global NER

During global NER, UvrA must recognize DNA damage, load UvrB onto the damaged DNA, and then dissociate from the preincision complex. Structural analysis of UvrA has aided in the identification of the regions responsible for interacting with DNA and with UvrB, and it has also revealed the architecture of the two ATPase sites (termed proximal and distal) within each UvrA monomer (Figure 1B) (Pakotiprapha et al., 2008, 2009; Timmins et al., 2009). The roles of these activities in global NER have been studied extensively, but their importance for TCR has not been investigated. As the process leading to the formation of the preincision complex is different in TCR and global NER, disruptive mutations within UvrA may fall into three classes: (1) mutations that affect a function that is equally important for global NER and TCR, (2) mutations that affect a function that is required for global NER but not for TCR, and (3) mutations that affect a function that is required for TCR but not for global NER.

We purified UvrA derivatives carrying substitutions that disrupt the proximal ATPase activity (K37A), the distal ATPase activity (K646A), DNA damage recognition (G502D, which likely exerts its effect indirectly), DNA binding activity (R712A R722A R724A R730A), and the UvrB interaction surface (R216A E222A) (Pakotiprapha et al., 2009; Thiagalingam and Grossman, 1991; Wang and Grossman, 1993) (Figure 1B). We also purified two deletion mutants: UvrA Δ131-248 lacks the UvrB binding domain (also likely to be the Mfd binding domain), and UvrA Δ290-400 lacks the “insertion domain” attached to the distal ATPase module (Pakotiprapha et al., 2008). To facilitate purification, the UvrA mutants carried an N-terminal His-tag, which has no effect on UvrA function in NER or TCR (Manelyte et al., 2009) (Figure S4). We checked the activity of the mutant proteins by measuring their GTPase activities, their interaction with UvrB (assessed by inhibition of GTPase activity), and their DNA binding activity (Table S1).

**Figure 6:**
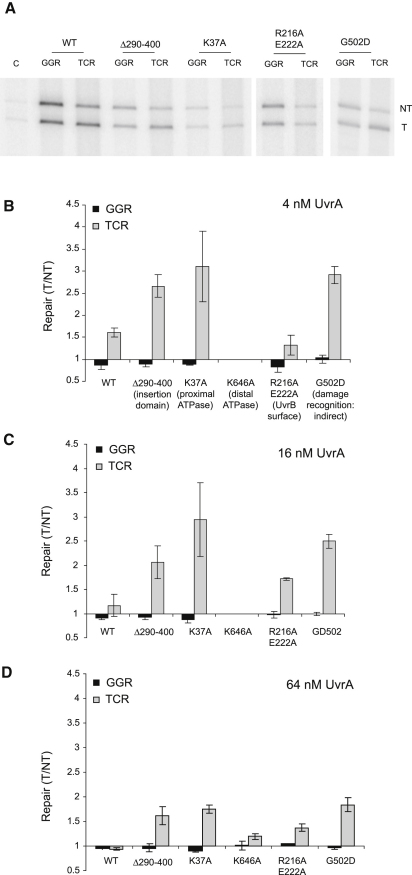
Effect of UvrA Derivatives on TCR In Vitro (A) TCR was analyzed using a patch-synthesis assay as described in Figure 3. Reactions contained 4 nM UvrA. (B–D) Quantification of relative repair of the template and nontemplate strands in reactions conducted with 4nM, 16 nM, or 64 nM UvrA. The figure shows the ratio of incorporation of radioactive label into the T and NT strands under the conditions indicated. Data are the mean of at least three independent experiments and are shown with standard deviation.

We tested the global NER and TCR activities of WT and mutant UvrA proteins in the patch-synthesis repair assay at concentrations of 4–64 nM (Figure 6). We found no mutants that could catalyze global NER but not TCR. Two of the mutant proteins (UvrA R712A R722A R724A R730A and UvrA Δ131-248) were unable to catalyze detectable global NER or TCR at any of the concentrations tested (data not shown), indicating that the UvrB-binding domain of UvrA and the ability to bind to DNA are essential for both pathways. UvrA K646A showed detectable levels of repair only at 64 nM, and preferential repair of the template strand was observed in the presence of Mfd and transcribing RNAP. Disruption of the distal ATPase activity thus decreases the efficiency of both global NER and TCR, but the distal ATPase is not essential for either process. UvrA R216A E222A showed a level of strand bias that was similar to the optimal level obtained with WT UvrA, indicating that the disruption of the UvrB/Mfd interaction surface affected global NER and TCR equally under the conditions of our assay.

Three of the mutant proteins (UvrA Δ290-400, UvrA K37A, and UvrA G502D) showed a significantly enhanced bias toward repair of the template strand under all conditions tested. As these mutants are all expected to cause loss of function, the simplest interpretation of these results is that these mutations have a greater detrimental effect on global NER (which repairs the nontemplate strand) than on TCR (which repairs the template strand). We conclude that the insertion domain, the proximal ATPase activity, and the DNA damage recognition activity of UvrA play a less important role in TCR than in global NER.

We also analyzed the ability of three mutant UvrB proteins to catalyze TCR. Each was defective in a function related to damage recognition. The proteins were UvrB K45A (containing a substitution in the Walker A box that abolishes ATPase activity [Seeley and Grossman, 1989]), UvrB Y95A Y96A (containing substitutions of residues at the base of the β-hairpin that are important for DNA damage recognition [Moolenaar et al., 2001]), and UvrB Y101A F108A (containing substitutions of residues at the tip of the β-hairpin that are important for the strand-separating/clamping activity of UvrB [Moolenaar et al., 2001]). In a patch-synthesis assay, none of these proteins supported detectable NER or TCR (data not shown). The absence of global NER activity is consistent with previous studies of these proteins, and the absence of TCR indicates that none of the properties of UvrB that are disrupted by the substitutions are made redundant by the action of RNAP and Mfd.

### The Insertion Domain of *E. coli* UvrA Is Involved in DNA Damage Recognition

**Figure 7:**
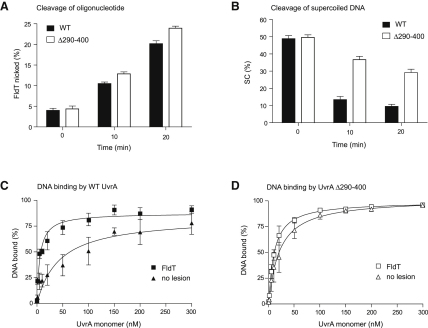
Activity of UvrA Δ290-400 in Incision and DNA Binding Assays (A) Nicking of a 50 bp duplex containing a single fluorescein (FldT) adduct. The substrate was incubated at 37°C with UvrA, UvrB, and UvrC, and aliquots were removed and quenched at the times indicated. The samples were analyzed by denaturing acrylamide gel electrophoresis, and the proportion of the radiolabeled DNA fragment that had been nicked was quantified. Data are the mean of three independent experiments and are shown with standard deviation. (B) Nicking of a UV-irradiated supercoiled plasmid. The plasmid was incubated at 37°C with UvrA, UvrB, and UvrC, and aliquots were removed and quenched at the times indicated. The samples were analyzed by native agarose gel electrophoresis, and nicking was detected as the conversion of supercoiled plasmid to nicked open circle DNA. Data are the mean of three independent experiments and are shown with standard deviation. (C and D) Binding of UvrA and UvrA Δ290-400 to a 50 bp duplex containing either a single FldT adduct or no lesion. The indicated concentrations of protein were incubated with 1 nM DNA for 20 min at 37°C. Samples were analyzed by EMSA. Data were fitted to a one-site binding equation, and the calculated dissociation constants were as follows: WT UvrA, K_d_^Fldt^ = 5.6 ± 1 nM, K_d_^no lesion^ = 43 ± 17 nM and UvrA Δ290-400, K_d_^Fldt^ = 10.5 ± 1 nM and K_d_^no lesion^ = 20.8 ± 4 nM. Data are the mean of three independent experiments and are shown with standard error.

A derivative of *Bacillus stearothermophilus* UvrA that lacked the insertion domain exhibited no apparent functional defects, and it was concluded that the insertion domain was not critical for UvrA function in vitro (Pakotiprapha et al., 2008). In contrast, analysis of *Deinococcus radiodurans* UvrA2 (a class II UvrA of uncertain function) suggested that its insertion domain is involved in DNA binding and damage recognition (Timmins et al., 2009). To understand why deletion of the insertion domain of *E. coli* UvrA (a class I UvrA) differentially affected TCR and global NER in our patch-synthesis assay, we examined the specificity of the DNA binding and global NER activities of UvrA Δ290-400. First, we analyzed the ability of UvrA Δ290-400 to support incision of a 50 bp duplex carrying a fluorescein-dT (FldT) adduct (Figure 7A). In agreement with the results of similar experiments by Pakotiprapha et al. (2008), we observed no difference between the activity of WT UvrA and UvrA Δ290-400 in this assay. We then analyzed the ability of UvrA Δ290-400 to support incision of UV-induced lesions in a supercoiled plasmid DNA template (Figure 7B). In contrast to the results with the short duplex, incision was significantly slower in the presence of UvrA Δ290-400 than in the presence of WT UvrA. UvrA Δ290-400 was also defective in the incision of UV-induced lesions from relaxed closed-circular plasmid templates, indicating that the topology of the template is not important for the effect (data not shown). We measured the affinity of WT UvrA and UvrA Δ290-400 for a 50 bp undamaged duplex and a 50 bp duplex containing a FldT adduct and found that deletion of the insertion domain decreased the ability of UvrA to distinguish between the two substrates (Figures 7C and 7D).

The reduced ability of UvrA Δ290-400 to catalyze global NER on the plasmid templates used in our patch-synthesis and incision assays likely results from the nonproductive binding of the protein to the undamaged DNA present in these templates, and we suggest that no defect was found in the NER activity of *B. stearothermophilus* UvrA lacking an insertion domain because the analysis was conducted with a short oligonucleotide that lacked nonspecific competitor DNA. We conclude that the insertion domain of *E. coli* UvrA is involved in damage recognition and that the model of DNA binding and damage recognition proposed for class II UvrAs (Timmins et al., 2009) is applicable to class I UvrAs.

## Discussion

When the product of the *mfd* gene was identified as the bacterial transcription-repair coupling factor, a striking feature was that a region toward the N-terminal region of the protein was similar in sequence to a region of UvrB (Selby and Sancar, 1993). It was found that this region of Mfd interacted with UvrA, and it has since been widely assumed that this interaction is responsible for the enhanced rate of repair that is observed during TCR (Hanawalt and Spivak, 2008; Roberts and Park, 2004; Selby and Sancar, 1993; Truglio et al., 2006a). In this work, we have tested this hypothesis experimentally. Our results show that the BHM of Mfd is essential for the Mfd-dependent enhancement of repair, although it is dispensable for the process by which RNAP is removed from the site of damage. The effect on TCR of deleting the BHM can be recapitulated in a full-length protein by substitution of conserved residues on the surface of D2 of the protein, and these substitutions disrupt the Mfd:UvrA interaction. Our results support the conclusion that the interaction of Mfd with UvrA is essential for TCR and that this interaction is similar in nature to the well-characterized UvrA:UvrB interaction.

In addition to its role in interaction with UvrA, the BHM of Mfd is also involved in autoregulation of the protein through its interaction with D7. The surface of D2 that interacts with UvrA overlaps with the surface that interacts with D7, and we found that substitution of residues within the area of overlap led to derepression of Mfd ATPase activity. Alanine substitutions of the residues within D7 that interact with D2 also led to derepression. The observation of disrupted autoregulation in these full-length variants of Mfd provides evidence that the interaction between D2 and D7 maintains the integrity of an inhibitory “clamp” that constrains the movement of the motor domains unless Mfd is bound to RNAP (Murphy et al., 2009). While the substitutions within D7 led to derepression of both ATPase activity and DNA translocase activity, the substitutions within D2 caused derepression of only ATPase activity. This suggests that the DNA translocase activity of Mfd can be regulated independently of the ATPase activity. It is possible that the substitutions made in D7 abolish the interdomain interaction, allowing the two halves of the inhibitory clamp to behave completely independently, whereas the substitutions made in D2 allow some interdomain movement at the interface without enabling the two parts of the inhibitory clamp to separate.

The finding that Mfd ΔD7 is able to catalyze TCR both in vitro and in vivo indicates that the regulatory mechanisms in which D7 is involved are not essential features of the TCR mechanism. Thus, although the autoinhibition of the DNA translocase activity is likely to benefit the cell by preventing nonproductive ATP hydrolysis and unscheduled displacement of RNAP, the mechanism by which repair is accelerated during TCR does not require a regulatable translocase activity. Similarly, while the ability of D7 to block the UvrA interaction surface in free Mfd appears to prevent sequestration of UvrA into nonproductive complexes (Deaconescu et al., 2006; Selby and Sancar, 1995a), the mechanism of TCR is not dependent on the ability of D7 to compete with UvrA for binding to the BHM.

To understand the mechanism by which the Mfd:UvrA interaction enhances the rate of repair, we searched for UvrA and UvrB mutants that were differentially affected in global NER and TCR. We found three UvrA mutants that were more defective in global NER than they were in TCR. The property that links the three is defective damage recognition. UvrA K37A carries a substitution within the Walker A motif of the proximal ATPase site that is thought to abolish ATP hydrolysis by this site without significantly affecting ATP binding (Thiagalingam and Grossman, 1991). One reported consequence of this substitution is that it abolishes the ability of UvrA to discriminate between UV-damaged and undamaged DNA (Thiagalingam and Grossman, 1991; Timmins et al., 2009). UvrA G502D is also unable to discriminate between UV-damaged and undamaged DNA (Wang and Grossman, 1993). It was originally studied because it lay within a putative DNA binding helix-turn-helix motif, but the crystal structure of *B. stearothermophilus* revealed that the residue is located well away from the likely path of the DNA and is likely to exert its effects on damage specificity indirectly (Pakotiprapha et al., 2008). UvrA Δ290-400 lacks the insertion domain, which is important for damage recognition in UvrA2 (Timmins et al., 2009). Our results show that the insertion domain is also important for DNA damage specificity in a class I UvrA of the type involved in NER and suggest an explanation for the fact that an earlier study found no defect in NER when UvrA lacked the insertion domain (Pakotiprapha et al., 2008). The differential effects of these three UvrA mutants on global NER and TCR indicates that Mfd, or a combination of Mfd and a stalled transcription complex, negates the need for UvrA to detect DNA damage in the way that it does during global NER. This in turn suggests that Mfd acts at the first step of the repair process, enhancing the rate of repair by altering the way in which UvrA loads onto the damaged DNA, rather than at a later stage such as destabilizing the UvrA:UvrB:DNA complex.

The discovery that the initial damage recognition step of bacterial NER (damage detection by UvrA) is dispensable for TCR raises parallels with eukaryotic repair pathways. The TCR and NER pathways in bacteria and eukaryotes follow broadly similar strategies but appear to have evolved independently (Ogrünç et al., 1998). The initial damage recognition step during global NER in eukaryotes involves the XPC protein, which is dispensable for TCR (Mu and Sancar, 1997). Our data now show that the involvement of RNAP and a transcription-repair coupling factor circumvents the need for mechanisms that are important for locating DNA lesions in nontranscribed DNA in both bacteria and eukaryotes.

## Experimental Procedures

### Strains, Plasmids, and Proteins

Details of strains, plasmids, and proteins used are given in Supplemental Experimental Procedures.

### Assays for DNA Translocation, RNAP Displacement, and NTPase Activity

DNA translocation by Mfd was assayed by monitoring the displacement of a triplex-forming oligonucleotide from supercoiled pSRTB1 plasmid as described in Smith et al. (2007). Displacement of stalled transcription complexes by Mfd was analyzed by EMSA as described in Smith et al. (2007), except that the DNA substrate carrying the T7A1 promoter was a PCR fragment generated from plasmid pSRT7A1 (Smith et al., 2007). ATPase activity was measured using an ATP-NADH-coupled spectrophotometric assay as described in Smith et al. (2007). GTPase activity was measured using the same protocol as ATPase activity, except that 2 mM GTP was used in place of ATP, NADH concentration was 200 μM, reaction volume was 500 μl, and absorbance was measured using a spectrophotometer. The concentrations of proteins used in GTPase assays are indicated in the relevant legends.

### In Vitro Patch-Synthesis Assay for TCR

Strand-specific repair was analyzed using a modification of the repair synthesis assay reported in Selby and Sancar (1993). A supercoiled DNA template containing randomly located UV-induced photoproducts was incubated for 20 min with UvrA, UvrB, UvrC, UvrD, DNA polymerase I, DNA ligase, dNTPs, rNTPs, NADH, and [α-^32^P]dATP. For TCR reactions, RNAP and Mfd were also present. The template contained an ∼170 bp reporter cassette downstream of the T7A1 promoter. Repair patches were detected by excising the reporter with BsrGI and SphI and analyzing the products by denaturing gel electrophoresis. Refer to Supplemental Experimental Procedures for full protocol.

### Primer Extension Analysis of TCR In Vivo

Strains AB1157 (*mfd^+^*) and UNCNOMFD (*mfd^−^*) transformed with pET21a (Novagen), pETMfd2, or pETMfd2 derivatives encoding mutant Mfd proteins were grown to mid-log phase, resuspended in M56 minimal salts, and irradiated with 40 J/m^2^ 254 nm UV light. Plasmid DNA was isolated at intervals, digested with RsaI, and analyzed by primer extension using Taq DNA polymerase and a radiolabeled primer that anneals to the template DNA strand from −104 to −85 upstream of the *lacI* gene carried on pET21a and pETMfd2. The products were analyzed by denaturing gel electrophoresis. Refer to Supplemental Experimental Procedures for full protocol.

### MFD Assay

Strains AB1157 (*mfd^+^ argE3(Oc)*) and UNCNOMFD (*mfd^−^ argE3(Oc)*) transformed with pET21a, pETMfd2, or pETMfd2 derivatives encoding mutant Mfd proteins were grown to mid-log phase, resuspended in M9 minimal medium that lacked amino acids, and irradiated with 20 J/m^2^ 254 nm UV light. Aliquots of irradiated culture were added to rich media either immediately or after 30 min incubation in M9 minimal medium that lacked amino acids. After overnight incubation, the frequency of Arg^+^ mutants in each sample was determined using selective growth media. Refer to Supplemental Experimental Procedures for full protocol.

### Bacterial Two-Hybrid Assay

Bacterial two-hybrid assays were performed as described in Manelyte et al. (2009) using reporter strain KS1 transformed with the indicated combinations of pRA02 and pRA03 derivatives encoding *rpoA-uvrA* and *cI-mfd* fusions.

### Assays for DNA Binding and Incision Activities of NER Proteins

DNA binding by UvrA was studied by EMSA. The substrates were blunt-ended ^32^P-labeled 50 bp duplexes with or without a single FldT adduct. UvrA proteins were incubated for 20 min at 37°C with 1 nM of substrate and 2 mM ATP. The ability of UvrA and UvrA Δ290-400 to support the steps of NER up to and including the point of incision by UvrC was assessed by monitoring the cleavage of either a ^32^P-labeled 50 nt oligonucleotide containing a single FldT adduct or a tritiated 4.2 kb ^3^H-labeled plasmid containing randomly located UV-induced lesions. Reactions contained DNA substrate, UvrA, UvrB, UvrC, and ATP and were incubated at 37°C. In each case, reaction products were analyzed by quantifying the labeled DNA following gel electrophoresis. Refer to Supplemental Experimental Procedures for full protocols.

## Supplemental Information

Document S1. Supplemental Experimental Procedures, Supplemental References, Four Figures, and One Table
