# Supplementary material for: Regulation and Rate Enhancement during Transcription-Coupled DNA Repair
Source: Mol Cell. 2010 Dec 10;40(5):714–24. doi: 10.1016/j.molcel.2010.11.012 (PMC3025350; doi:10.1016/j.molcel.2010.11.012)
Supplement: Document S1. Supplemental Experimental Procedures, Supplemental References, Four Figures, and One Table [file mmc1.pdf]

Supplemental Information
Molecular Cell, *Volume 40*

Regulation and Rate Enhancement during Transcription-Coupled DNA Repair

Laura Manelyte, Young-In T. Kim, Abigail J. Smith, Rachel M. Smith, and Nigel J. Savery

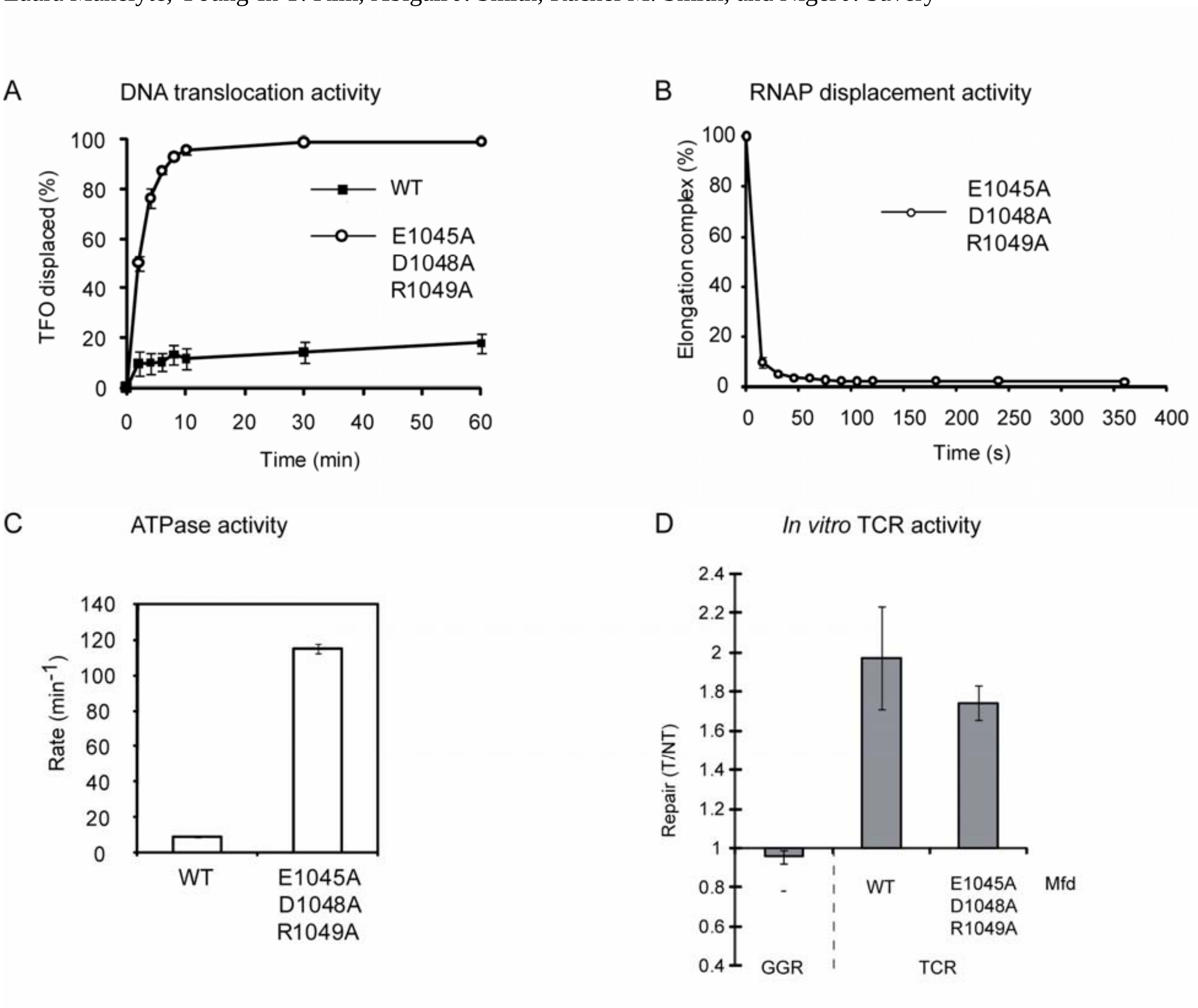

Figure S1 (related to Figure 2). Biochemical Analysis of Mfd E1045A D1048A R1049A

(A) DNA translocation activity. Displacement of a triplex-forming oligonucleotide (TFO) from supercoiled pSRTB1 plasmid was monitored by EMSA. The graph shows the percentage of TFO displaced at each time point, normalized for the amount of triplex present at t=0. Data are the mean of 3 independent experiments, and are shown with SD.

(B) RNAP displacement activity. Transcription complexes were stalled on a linear DNA fragment carrying the T7A1 promoter by nucleotide starvation and the addition of 3′ dUTP. Displacement by Mfd was monitored by EMSA. The graph shows the percentage of transcription elongation complex displaced at each time point. Data are the mean of 3 independent experiments, and are shown with SD.

(C) ATPase activity. Rates were measured at 37°C using an NADH-coupled assay at 2 mM ATP. Data are the mean of two independent experiments, and are shown with range.

(D) In vitro TCR activity. TCR was analyzed using a patch synthesis assay as described in Figure 3. TCR reactions contained RNAP, UvrA, B, C, D, DNA ligase, DNA polymerase and, Mfd or Mfd E1045A D1048A R1049A as indicated. Global NER reactions (GGR) were performed as for TCR reactions, except that RNAP and Mfd were omitted. The figure shows the ratio of incorporation of radioactive label into the T and NT strands. Data are the mean of 3 independent experiments, and are shown with SD.

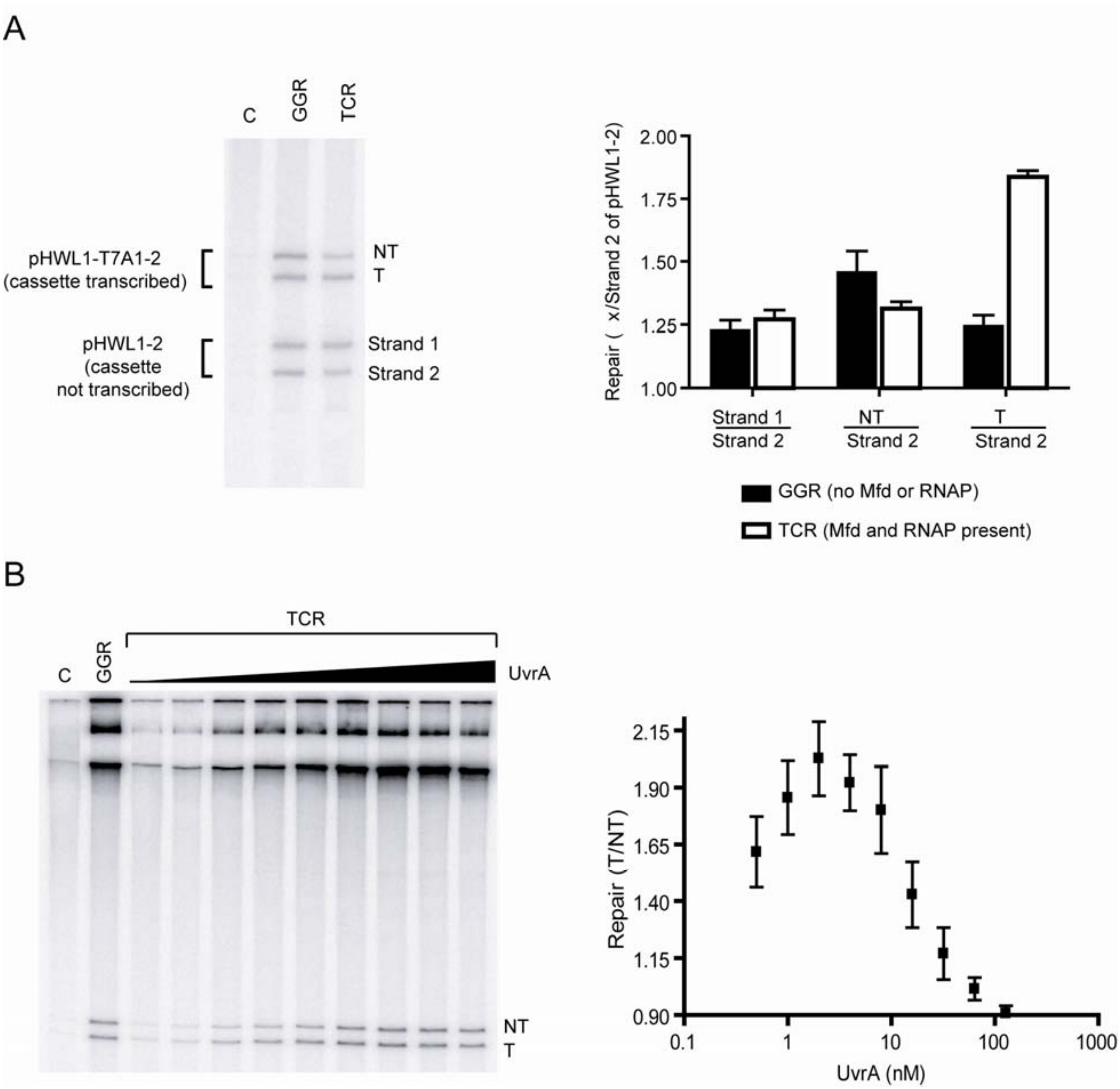

Figure S2 (related to Figure 3). Dependence of TCR Reactions on UvrA Concentration and Comparison of TCR with GGR

(A) Comparison of the repair of transcribed DNA and nontranscribed DNA in vitro. This experiment was conducted to verify the use of the nontemplate strand of the pHWL1-T7A1-2 reporter cassette as an internal measure of global NER in in vitro TCR assays. TCR was analyzed using a patch synthesis assay essentially as described in Figure 3. Reactions contained 1.5 nM UV-irradiated plasmid pHWL1-T7A1-2 (which contains a reporter cassette downstream of the T7A1 promoter), and also 1.5 nM UV-irradiated

plasmid pHWL1-2 (which contains a shorter reporter cassette that is not transcribed). The experiment allowed repair patch synthesis in the T7A1 transcription unit during TCR to be directly compared with repair patch synthesis by global NER in nontranscribed pHWL1-2 DNA. Cleavage of the plasmid mixture by BsrGI and SphI produced nontemplate strand (NT) and template strand (T) fragments from pHWL1-T7A1-2, and two shorter strands from pHWL1-2 (labeled strands 1 and 2). TCR reactions contained RNAP, UvrB, C, D, DNA ligase, DNA polymerase, Mfd and 8 nM UvrA. Global NER reactions (GGR) were performed as for TCR reactions, except that RNAP and Mfd were omitted. The control reaction in lane C was performed as for the GGR reaction except that UvrA, B, C and D were omitted. The gel image shows the incorporation of [α-^{32}P]dATP into repair patches in the four separate strands under the conditions specified. The chart shows the intensities of strand 1 (pHWL1-2) and strands T and NT (pHWL1-T7A1-2) normalized to the intensity of strand 2 (pHWL1-2). The intensity of strand 1 relative to strand 2 is the same in TCR and GGR reactions as both strands of this nontranscribed reporter are repaired by global NER regardless of the conditions. The intensity of strand T relative to strand 2 increases under TCR conditions, because strand T is a substrate for the TCR pathway and its repair is enhanced by RNAP and Mfd. The intensity of strand NT relative to strand 2 does not differ significantly between GGR and TCR reactions. This result confirms that, under TCR conditions, patch synthesis in the nontemplate strand of pHWL1-T7A1-2 represents global NER. Data are the mean of a 3 independent experiments and are shown with SD.

(B) Effect of UvrA concentration on the strand-bias of TCR in vitro. TCR was analyzed using a patch synthesis assay as described in Figure 3. The gel image shows the incorporation of [α-^{32}P]dATP into repair patches in the template (T) and nontemplate (NT) strand of a reporter cassette located downstream of the T7A1 promoter. TCR reactions contained RNAP, UvrB, C, D, DNA ligase, DNA polymerase and Mfd, and 0.5, 1, 2, 4, 8, 16, 32, 64 and 128 nM UvrA. GGR reactions contained 4 nM UvrA, and were performed as for TCR reactions except that RNAP and Mfd were omitted. The control reaction in lane C was performed as for the GGR reaction except that UvrA, B, C and D

were omitted. The chart shows the ratio of incorporation of radioactive label into the T and NT strands. The intercept of the x axis on the y axis denotes the ratio of repair obtained in the GGR reactions. Data are the mean of 3 independent experiments, and are shown with SD.

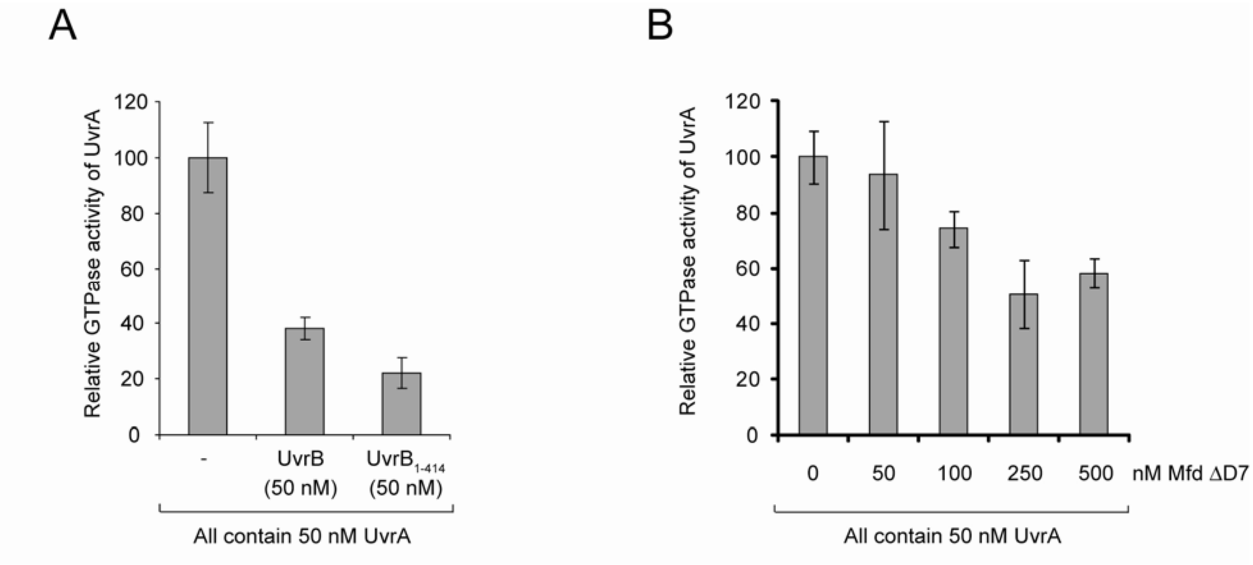

Figure S3 (related to Figure 5). Effect of UvrB and Mfd Truncations on the GTPase Activity of UvrA

Assays contained 50 nM UvrA, and the indicated concentrations of UvrB or Mfd proteins. Rates were measured at 37°C using an NADH-coupled assay and 2 mM GTP. Data are the mean of 3 independent experiments, and are shown with SD.

(A) A fragment of UvrB that contained only domains 1a, 2 and 1b ($UvrB_{1\text{-}414}$) decreased the GTPase activity of UvrA as effectively as full-length UvrB did.

(B) Effect of Mfd ΔD7 concentration on the inhibition of UvrA GTPase activity.

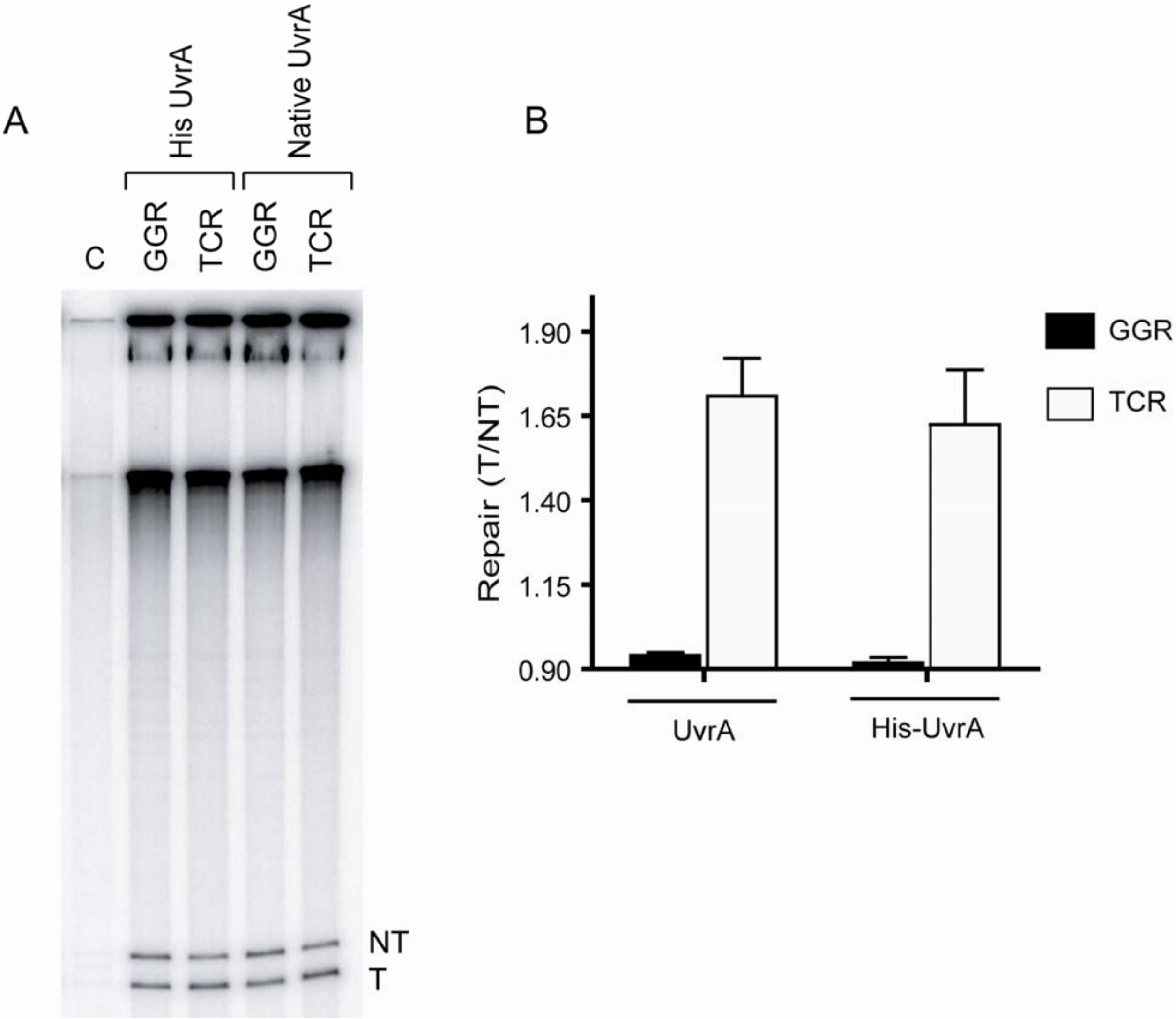

Figure S4 (related to Figure 6). Comparison of Native and His-Tagged UvrA

TCR was analyzed using a patch synthesis assay as described in Figure 3.

(A) The figure shows the incorporation of [α-^{32}P]dATP into repair patches in the template (T) and nontemplate (NT) strand of a reporter cassette located downstream of the T7A1 promoter. TCR reactions contained RNAP, UvrB, C, D, DNA ligase, DNA polymerase and Mfd, and 4 nM native or His-tagged UvrA as indicated. GGR reactions were performed as for TCR reactions except that RNAP and Mfd were omitted. The control reaction in lane C was performed as for the GGR reaction except that UvrA, B, C and D were omitted.

(B) Quantification of relative repair of the template and nontemplate strands. The figure shows the ratio of incorporation of radioactive label into the T and NT strands. Data are the mean of three independent experiments and are shown with SD.

Table S1 (related to Figure 6). Biochemical Analysis of UvrA Derivatives

Region affected	Mutant	Rate of GTP hydrolysis (min^{-1})		DNA binding
		-UvrB	+UvrB	
-	WT	71±7	27±5	+
Proximal ATPase	K37A	8.4±2	13±2	+
Distal ATPase	K646A	36±3	38±5	ND
Insertion domain	Δ290-400 (GT)	57±2	27±6	+
Damage recognition (indirect)	G502D	67±2	44±7	+
DNA binding	R712A R722A R724A R730A	38±2	30±6	-
UvrB binding domain	Δ131-248 (GT)	74±1	67±3	ND
UvrB/Mfd binding surface	R216A E222A	77±11	68±11	ND

Rates of GTP hydrolysis by UvrA were measured at 37°C using an NADH-coupled assay and 2 mM GTP. Assays contained 50 nM of each UvrA protein (except UvrA K37A, which was present at 100 nM). Where indicated, UvrB was present at 50 nM (except in the UvrA K37A plus UvrB reactions, which contained 100 nM UvrB). Data are the mean of 3 independent experiments, and are shown with SD. DNA binding properties of UvrA derivatives were determined by EMSA. UvrA derivatives at concentrations from 1-800 nM were incubated with 1 nM radiolabeled 50 bp duplex containing a single FldT adduct for 20 min at 37°C before analysis. ("+" DNA binding was detected at < 300 nM UvrA; "-" No DNA binding detected at 800 nM UvrA.; "ND" not determined). As shown previously, the substitutions within either the proximal ATPase site or the distal ATPase site decreased the NTPase activity of the protein (Myles et al., 1991; Thiagalingam and Grossman, 1991). UvrB did not affect the residual activity of either mutant. UvrA R712A R722A R724A R730A showed a reduced GTPase activity that was not affected by UvrB, and was unable to bind to DNA even at high protein

concentration. The GTPase activities of the remaining mutants were all close to WT, but differed in their response to UvrB: UvrB repressed the activity of UvrA Δ290-400 and UvrA G502D, but did not repress the activity of UvrA Δ131-248 (which lacks the UvrB binding domain) or UvrA R216A E222A (in which the UvrB interaction surface is disrupted).

SUPPLEMENTAL EXPERIMENTAL PROCEDURES

Plasmid and Strain Construction

pETMfd-T7 encodes untagged Mfd under the control of a T7 promoter. Because *mfd* contains an internal NdeI site the plasmid was constructed in two stages: (i) the NdeI – HindIII fragment of pETMfd2 (Deaconescu et al., 2006) encoding residues 911 onwards was cloned into the corresponding sites of pET21a (Novagen) to create an intermediate plasmid; (ii) the *mfd* gene was amplified by PCR using a primer that introduced an NdeI site upstream of the start codon, and the product was digested with NdeI and ligated into the NdeI site of the intermediate construct. pETMfd-T7 E1045A D1048A R1049A was created from pETMfd-T7 by Quikchange mutagenesis (Stratagene). pETMfdΔBHM-T7 encodes untagged Mfd ΔBHM (Mfd residues 354-1148) under the control of a T7 promoter: the *mfd* gene was amplified by PCR using a primer that introduced an NdeI site and a start codon upstream of codon 354, and the product was digested with NdeI and ligated between the NdeI sites of pETMfd-T7. pMfd19 R165A R181A F185A encodes an untagged Mfd derivative under the control of the *mfd* promoter, and was created from pMfd19 (Selby and Sancar, 1993) by "rolling circle PCR" mutagenesis (Kirsch and Joly, 1998). pETMfd2 R165A R181A F185A also encodes an untagged Mfd derivative under the control of the *mfd* promoter, and was created by transferring NdeI-NdeI fragments from pMfd19 R165A R181A F185A into NdeI-digested pETMfd2 (Deaconescu et al., 2006). pETMfd2 ΔBHM encodes untagged Mfd ΔBHM under the control of the *mfd* promoter: a region of the *mfd* gene spanning the two natural NdeI sites (located 41 bp upstream of the start codon and at codon 910) was amplified by PCR using an upstream primer that deleted codons 2-353, and the product was

digested with NdeI and ligated between the NdeI sites of pETMfd2. pETMfd2ΔD7 has been described previously (Deaconescu et al., 2006). pETMfd-T7 R165A R181A F185A ΔD7 encodes untagged Mfd ΔD7 carrying R165A R181A F185A substitutions, under the control of a T7 promoter: the mutated *mfd* gene was amplified from pETMfd2 R165A R181A F185A using a primer that inserted an NdeI site upstream of the start codon, and an NdeI-NdeI fragment was transferred into NdeI-digested pETMfd$_{1\text{-}997}$-T7 (Smith et al., 2007).

pETDuetUvrA encodes UvrA under the control of a T7 promoter, and was created in two stages: (i) the *uvrA* gene was amplified from MG1655 genomic DNA using primers that introduced a BamHI site immediately upstream of the start codon, an NcoI site overlapping the start codon, and a HindIII site immediately downstream of the stop codon; the PCR fragment was ligated as a BamHI – HindIII fragment into the corresponding sites in the polylinker of pBluescript II KS(-) (Stratagene) to create pBSIIUvrA; (ii) the *uvrA* gene was excised from pBSIIUvrA as an NcoI – HindIII fragment and ligated into the corresponding sites in pETDuet-1 (Novagen). Expression vectors encoding mutant N-terminally His-tagged UvrA or UvrB proteins carrying substitutions or deletions, were derived from pQE30UvrA and pQE30UvrB respectively (Manelyte et al., 2009), using "rolling circle PCR" mutagenesis (Kirsch and Joly, 1998). In pQE30 Δ131-248 and pQE30UvrA Δ205-400 the deleted residues were replaced with a G-T dipeptide, as in (Pakotiprapha et al., 2008).

pRA02 UvrA$_{1\text{-}252}$ encodes residues 1-252 of UvrA fused to the C-terminus of residues 1-248 of the RNAP α subunit: the internal KpnI site within the *uvrA* gene of pBSIIUvrA was destroyed by silent mutagenesis to create pBSIIUvrAΔKpn; this was used as a template for PCR amplification by primers that placed an XbaI site upstream of codon 1 and a KpnI site downstream of codon 252; the PCR product was digested with XbaI and KpnI and ligated into the corresponding sites in pRA02 (Manelyte et al., 2009). pRA03 (Manelyte et al., 2009) derivatives encoding residues 1-219 of WT Mfd and Mfd D2AAA (R165A R181A F185A) fused to the C-terminus of λcI were constructed by PCR amplification from the appropriate pETMfd2 derivatives using primers that placed an XbaI site upstream of codon 1, and a stop codon and KpnI site downstream of codon

219. The resulting PCR products were digested with XbaI and KpnI and ligated into the corresponding sites in pRA03.

To create pHWL1-T7A1-2 the 1165 bp BsrGI to HindIII luciferase gene fragment from pRCB-KA4 (Deaconescu et al., 2006) was ligated into the HindIII site of pSRT7A1 (Smith et al., 2007) after all DNA ends had been filled in using Klenow polymerase and dNTPs. The resulting plasmid contains a BsrGI site centred at +39.5 wrt the T7A1 transcription start site and an SphI site centred at +208.5. Digestion with BsrGI and SphI yields a fragment in which the template strand is 165 nucleotides long and the nontemplate strand is 173 nucleotides long. To create pHWL1-2, pHWL1-T7A1-2 was digested with EcoRI and BsrGI to remove the promoter. The resulting 5' overhangs were converted to blunt ends by the addition of Klenow polymerase and dNTPs, and the plasmid backbone was religated to generate pHWL1-NP. pHWL1-NP was then digested with MfeI and BstEII, excising a fragment from the reporter cassette. The resulting 5' overhangs were converted to blunt ends by the addition of Klenow fragment and dNTPs. The plasmid backbone was religated to generate pHWL1-2. pHWL1-2 contains a reporter cassette that is not transcribed, and digestion with BsrGI and SphI yields a fragment comprised of strands 141 and 133 nucleotides long. FB21635 is a derivative of *E. coli* strain MG1655 carrying a *Tn5*(Kan^R) insertion in the chromosomal *uvrA* gene (Kang et al., 2004). The strain was cured of the temperature-sensitive pKD46 plasmid by growth at 43°C, and was then transformed with the F´ plasmid from XL1-Blue (Stratagene) as described by (Miller, 1972). The resulting strain is FB21635 [F´ *proAB* $lacI^qZ\Delta M15$ Tn*10* (Tet^R)]. JW0762-2 is a derivative of *E. coli* strain BW25113 carrying a Kan^R-cassette that disrupts the chromosomal *uvrB* gene (Baba et al., 2006). The strain was transformed with the F´ plasmid from XL1-Blue (Stratagene) as described by (Miller, 1972), to create strain JW0762-2 [F´ *proAB* $lacI^qZ\Delta M15$ Tn*10* (Tet^R)].

Proteins

All protein concentrations refer to monomers. His-tagged RNAP was purified as described in (Smith and Savery, 2005). His-tagged WT UvrB, His-tagged $UvrB_{1-414}$, His-tagged UvrC, and untagged UvrD were purified as described in (Manelyte et al., 2009).

Mfd ΔD7 was purified as described in (Smith et al., 2007). *E. coli* DNA ligase, DNA polymerase I and restriction enzymes were purchased from New England Biolabs.

WT Mfd was purified from BL21(DE3) cells transformed with pETMfd-T7: cells were grown in LB containing appropriate antibiotics at 37°C to mid-log phase (A_{600} ~0.5), expression was induced by the addition of 1 mM IPTG and the culture was incubated for 3 hr at 37°C before harvesting. Mfd ΔBHM was purified from BB834(DE3) cells transformed with pETMfdΔBHM-T7: cells were grown in LB containing appropriate antibiotics at 37°C to mid-log phase (A_{600} ~0.5), expression was induced by the addition of 1 mM IPTG and the culture was incubated for 1 hr 40 min at 30°C before harvesting. Mfd E1045A D1048A R1049A was purified from BL21(DE3) *Δmfd* cells transformed with pETMfd-T7 E1045A D1048A R1049A. BL21(DE3) *Δmfd* carries the *mfd*$^-$ allele from UNCNOMFD (Selby and Sancar, 1995), (A. Yuan and A. Hochschild, unpublished). Cells were grown in LB containing appropriate antibiotics at 37°C to mid-log phase (A_{600} ~0.5), expression was induced by the addition of 1 mM IPTG and the culture was incubated for 3 hr at 30°C before harvesting. Mfd R165A R181A F185A (Mfd D2AAA) was purified from FB20256 (a derivative of MG1655 carrying a *Tn5*(Kan^R) insertion in the chromosomal *mfd* gene (Kang et al., 2004)) transformed with pMfd19 R165A R181A F185A: cells were grown in LB containing appropriate antibiotics at 37°C until the A_{600} was 1.5, and were then harvested. Mfd R165A R181A F185A ΔD7 (Mfd D2AAA ΔD7) was purified from BL21(DE3) *Δmfd* cells transformed with pETMfd-T7 R165A R181A F185A ΔD7: cells were grown in LB containing appropriate antibiotics at 37°C to mid-log phase (A_{600} ~0.5), expression was induced by the addition of 1 mM IPTG and the culture was incubated for 1 hr at 27°C before harvesting. In all cases the untagged Mfd proteins were purified from the harvested cell pellet as described in Chambers et al., 2003.

Untagged WT UvrA (used for experiments presented in Figures 3, S1D, S2, S3A and S4) was purified from BL21(DE3) pLysS transformed with pETDuetUvrA: cells were grown in LB containing appropriate antibiotics at 37°C to mid-log phase (A_{600} ~0.5), expression was induced by the addition of 1 mM IPTG and the culture was incubated for 1 hr at 37°C before harvesting. UvrA was purified from the pellet using HiTrap blue

sepharose, heparin and MonoQ columns (all GE Healthcare) using the procedures and buffers described for Mfd purification in (Chambers et al., 2003). His-tagged WT and mutant UvrA proteins (used for experiments presented in Figures 5A, 6, 7, S3B, S4 and Table S1) were purified from FB21635 [F´ *proAB lacI*^{q}ZΔ*M15* Tn*10* (Tet^{R})] transformed with the appropriate pQE30UvrA derivatives, following the procedure described in (Manelyte et al., 2009). His-tagged mutant UvrB proteins were purified from JW0762-2 [F´ *proAB lacI*^{q}ZΔ*M15* Tn*10* (Tet^{R})] transformed with the appropriate pQE30UvrB derivatives, following the procedure described in (Manelyte et al., 2009), except that cultures were grown at 30°C.

In Vitro Patch Synthesis Assay for TCR

Strand-specific repair was analyzed using a modification of the repair synthesis assay reported in (Selby and Sancar, 1993). Supercoiled DNA templates containing randomly located UV-induced photoproducts were generated by irradiating plasmid DNA in 20 µl 15 nM aliquots with 30 J/m^{2} 254 nm UV light. Reactions (20 µl) were performed in TK buffer (40 mM HEPES pH 8.0, 8 mM $MgCl_2$, 50 mM KCl, 5 mM DTT, 4% glycerol and 100 µg/ml BSA) supplemented with 2 mM ATP, 10 µM dATP, 100 µg/ml NADH and 200 µM each of dGTP, dCTP, dTTP, GTP, CTP and UTP. Unless stated otherwise, all GGR and TCR reactions contained 4 nM UvrA, 100 nM UvrB, 70 nM UvrC, 5 nM UvrD, 1.25 U/ml *E. coli* DNA polymerase I and 24 U/ml *E. coli* DNA ligase. Where indicated, 20 nM RNAP and 250 nM WT or mutant Mfd were present. Unless stated otherwise, reactions were started by the addition of UV-irradiated pHWL1-T7A1-2 DNA at a final concentration of 1.5 nM, together with 5 µCi [α-^{32}P]dATP (3000 Ci/mmol). Reactions were incubated at 37°C for 20 min, then stopped by phenol/chloroform extraction. The extracted DNA was ethanol-precipitated, and each DNA pellet was resuspended in 50 µl NEB buffer 2 + 100 µg/ml BSA, and digested with 5 U BsrGI and 5 U SphI. The digested DNA was purified by phenol/chloroform extraction and ethanol precipitation. Dried DNA pellets were resuspended in denaturing loading dye (95% formamide, 20 mM EDTA, 0.25% xylene cyanol and 0.25% bromophenol blue), incubated at 90°C for 4 min and then analyzed by denaturing gel electrophoresis (6% polyacylamide/7M Urea/1x TBE gels). After electrophoresis gels were fixed with 10% acetic acid/10%

methanol, dried and analyzed using a Molecular Dynamics Typhoon PhosphorImager and ImageQuant software.

Primer Extension Analysis of TCR In Vivo

Strain UNCNOMFD (*mfd*$^-$) (Selby and Sancar, 1995) was transformed with pET21a (Novagen), pETMfd2, or pETMfd2 derivatives encoding mutant Mfd proteins. Strain AB1157 (*mfd*$^+$) was transformed with pET21a. Each strain was grown to mid-log phase (A_{600} 0.35 – 0.4) in LB supplemented with 100 μg/ml ampicillin. 50 ml of culture was harvested by centrifugation, and the cells were resuspended in 50 ml M56 minimal salts. The resuspended culture was irradiated with 40 J/m^2 254 nm UV light, using a Petri dish with a surface area of 520 cm^2. Aliquots of culture were removed before and directly after UV-irradiation and placed on ice. The remaining culture was transferred into a foil-wrapped flask, supplemented with 0.25% casamino acids, 0.4% glucose and 4 μg/ml thiamine and incubated in the dark at 37°C with shaking. At intervals 15 ml aliquots were removed and placed on ice. Plasmid DNA was isolated from each 15 ml aliquot using a Qiagen spin miniprep kit. One third of the purified DNA was digested with 3 U RsaI in NEB buffer 2. This digest releases a fragment that extends from -457 to +526 wrt the *lacI* transcription start site. The complete digest was used without further purification as the template for a primer extension reaction containing 80 nM primer that had been end-labeled using [γ-^{32}P]ATP (5'-CCT ACT ACT GGG CTG CTT CCT AAT G-3': anneals to the template DNA strand from -104 to -85 upstream of the *lacI* gene carried on pET21a and pETMfd2), 400 μM dNTPs, 0.05 U/μl *Taq* polymerase (Fermentas) and 1x $(NH_4)_2SO_4$ Taq buffer (Fermentas). The reactions were incubated for 5 cycles of [94°C 1 min, 60°C 1 min, 72°C 3 min]. The DNA was purified by phenol/chloroform extraction and ethanol precipitation. Dried DNA pellets were resuspended in denaturing loading dye, and then analyzed as described for the repair-patch synthesis assay.

Assay for Mutation Frequency Decline

Mutation frequency decline was monitored using an assay based on that described in (Fabisiewicz and Janion, 1998). The strains used were AB1157 (relevant genotype *mfd*$^+$

argE3(Oc)), and UNCNOMFD (*mfd*⁻ derivative of AB1157) transformed with pETMfd2 derivatives or pET21a. In experiments with UNCNOMFD transformants the liquid LB and agar plates used were supplemented with 100 µg/ml ampicillin. Cultures were grown in LB until the A_{600} was approximately 0.3. The cultures were then harvested by centrifugation and the pellets were resuspended at an A_{600} of approximately 0.3 in M9 medium that lacked amino acids and glucose. Each resuspended culture was warmed to 37°C, and then a 5 ml aliquot was placed in a 90 mm diameter Petri dish and irradiated with 20 J/m^2 254 nm light. The irradiated culture was immediately supplemented with glucose (0.4% w/v final concentration), and a 1 ml sample was withdrawn and added to 5 ml LB that had been prewarmed to 37°C (zero time sample). The remaining irradiated culture was incubated at 37°C for 30 min, and then a second 1 ml sample was withdrawn and added to 5 ml LB that had been prewarmed to 37°C (30 min. sample). The two samples were incubated overnight at 37°C. The overnight cultures were serially diluted in M9 medium lacking amino acids and glucose, and appropriate dilutions were plated on LB agar (to determine cfu per ml) and M9 agar supplemented with 0.4% w/v glucose, and 25 µg/ml histidine, leucine, proline and threonine (to identify Arg^+ mutants). Plates were incubated at 37°C before counting and determining the frequency of reversion to Arg^+. All UV-induced mutation frequencies measured were between 1×10^{-6} and 3×10^{-5}.

Assays for DNA Binding and Incision Activities of NER Proteins

DNA binding by UvrA was studied by EMSA. The substrates were blunt-ended ^{32}P-labeled 50 bp duplexes with or without a single FldT adduct 29 bp from the 5′ end of one strand (sequence as described for incision assays). UvrA proteins were incubated for 20 min at 37°C with 1 nM of substrate, in repair buffer (40 mM Hepes pH 8.0, 100 mM KCl, 8 mM $MgCl_2$, 4% glycerol (v/v), 5 mM DTT, 100 µg/ml BSA) supplemented with 2 mM ATP and an ATP regeneration system (0.012 U/µl pyruvate kinase, 0.018 U/µl lactate dehydrogenase and 1 mM phosphoenol pyruvate). Samples were run on a 4.5% native polyacrylamide gel in 1x TBE at 10.8 V/cm for 1 hr at 4°C. Gels were dried and analyzed using a Molecular Dynamics Typhoon PhosphorImager and ImageQuant and Graphpad Prism software.

The ability of UvrA and UvrA Δ290-400 to support the steps of NER up to and including the point of incision by UvrC was assessed in two ways. To monitor incision at a FldT lesion a 50 nt oligonucleotide containing a FldT adduct (5'-CTA GGA TCG GAT AGC GCA TGA CAG TGA C FldT G GAT CGA CAG CGT CGT ATG AG-3') was labeled with ^{32}P and annealed to an undamaged oligonucleotide of complementary sequence. Reactions contained 4 nM DNA substrate, 100 nM UvrB, 70 nM UvrC and 2 mM ATP in TK buffer. The reaction was started by the addition of UvrA or UvrA Δ290-400 at a final concentration of 4 nM, and was incubated at 37°C. Samples were removed at the indicated times, and mixed with an equal volume of denaturing loading dye. The samples were incubated at 95°C for 5 min, then analyzed by denaturing gel electrophoresis (10% polyacrylamide/7M Urea/1x TBE gels). Gels were dried, and analyzed using a Molecular Dynamics Typhoon PhosphorImager, and ImageQuant software. To monitor incision at UV-induced lesions a supercoiled DNA template containing randomly located UV-induced photoproducts was generated by irradiating a tritiated 4.2 kb ^{3}H-labeled plasmid (Bellamy et al., 2007) in 20 µl 17 nM aliquots with 30 J/m^2 254 nm UV light. The UV-irradiated plasmid DNA (4 nM) was mixed with 100 nM UvrB, 70 nM UvrC and 2 mM ATP in TK buffer. The reactions were started by the addition of UvrA at a final concentration of 4 nM and incubated at 37°C. The reactions were stopped by the addition of 0.25x volume of 5x STEB (100 mM Tris-HCl pH 8.0, 100 mM EDTA pH 8.0, 1.17 M sucrose, 0.4 mg/ml bromphenol blue). Samples were analyzed on 1% agarose gels in 1x TAE and the bands corresponding to supercoiled and nicked DNA in each sample were excised from the gel and quantified by scintillation counting.

RNAP-Displacement Assays

The RNAP-displacement assays presented in Figure S2 used a slightly modified version of the protocol described in (Smith and Savery, 2005; Smith et al., 2007). The DNA substrate used was a 570 bp PCR fragment generated from plasmid pAR1707 (Levin et al., 1987). This fragment contains the T7A1 promoter and is essentially the same as the RsaI/SmaI fragment of pAR1707 described in (Smith and Savery, 2005). Transcription initiation complexes were formed on this fragment by incubating 0.4 nM end-labeled

DNA with 10 nM RNAP holoenzyme in repair buffer for 15 min at 37°C. Heparin was added at a final concentration of 10 µg/ml and reactions were incubated at 37°C for a further 15 min. To form elongation complexes stalled at +21 ApU (100 µM), ATP (10 µM), CTP (10 µM), GTP (10 µM), 3'dUTP (10 µM) and dATP (2 mM) were added and the reaction mixtures were incubated at 37°C for 15 min. Mfd or its derivatives were added at 250 nM and the reactions were incubated at 37°C. At timed intervals, aliquots were removed and analyzed by EMSA as described in (Smith et al., 2007).

SUPPLEMENTAL REFERENCES

Baba, T., Ara, T., Hasegawa, M., Takai, Y., Okumura, Y., Baba, M., Datsenko, K.A., Tomita, M., Wanner, B.L., and Mori, H. (2006). Construction of Escherichia coli K-12 in-frame, single-gene knockout mutants: the Keio collection. Mol Syst Biol *2*.

Bellamy, S.R., Milsom, S.E., Kovacheva, Y.S., Sessions, R.B., and Halford, S.E. (2007). A switch in the mechanism of communication between the two DNA-binding sites in the SfiI restriction endonuclease. J Mol Biol *373*, 1169-1183.

Chambers, A.L., Smith, A.J., and Savery, N.J. (2003). A DNA translocation motif in the bacterial transcription-repair coupling factor, Mfd. Nucleic Acids Res *31*, 6409-6418.

Deaconescu, A.M., Chambers, A.L., Smith, A.J., Nickels, B.E., Hochschild, A., Savery, N.J., and Darst, S.A. (2006). Structural basis for bacterial transcription-coupled DNA repair. Cell *124*, 507-520.

Fabisiewicz, A., and Janion, C. (1998). DNA mutagenesis and repair in UV-irradiated E. coli K-12 under condition of mutation frequency decline. Mutat Res *402*, 59-66.

Kang, Y., Durfee, T., Glasner, J.D., Qiu, Y., Frisch, D., Winterberg, K.M., and Blattner, F.R. (2004). Systematic mutagenesis of the Escherichia coli genome. J Bacteriol *186*, 4921-4930.

Kirsch, R.D., and Joly, E. (1998). An improved PCR-mutagenesis strategy for two-site mutagenesis or sequence swapping between related genes. Nucleic Acids Res *26*, 1848-1850.

Levin, J.R., Krummel, B., and Chamberlin, M.J. (1987). Isolation and properties of transcribing ternary complexes of *Escherichia coli* RNA polymerase positioned at a single template base. J Mol Biol *196*, 85-100.

Manelyte, L., Guy, C.P., Smith, R.M., Dillingham, M.S., McGlynn, P., and Savery, N.J. (2009). The unstructured C-terminal extension of UvrD interacts with UvrB, but is dispensable for nucleotide excision repair. DNA Repair *8*, 1300-1310.

Miller, J. (1972). In Experiments in Molecular Genetics (Cold Spring Harbor Laboratory Press).

Myles, G.M., Hearst, J.E., and Sancar, A. (1991). Site-specific mutagenesis of conserved residues within Walker A and B sequences of Escherichia coli UvrA protein. Biochemistry *30*, 3824-3834.

Pakotiprapha, D., Inuzuka, Y., Bowman, B.R., Moolenaar, G.F., Goosen, N., Jeruzalmi, D., and Verdine, G.L. (2008). Crystal structure of Bacillus stearothermophilus UvrA provides insight into ATP-modulated dimerization, UvrB interaction, and DNA binding. Mol Cell *29*, 122-133.

Selby, C.P., and Sancar, A. (1993). Molecular mechanism of transcription-repair coupling. Science *260*, 53-58.

Selby, C.P., and Sancar, A. (1995). Structure and function of transcription-repair coupling factor. I. Structural domains and binding properties. J Biol Chem *270*, 4882-4889.

Smith, A.J., and Savery, N.J. (2005). RNA polymerase mutants defective in the initiation of transcription-coupled DNA repair. Nucleic Acids Res *33*, 755-764.

Smith, A.J., Szczelkun, M.D., and Savery, N.J. (2007). Controlling the motor activity of a transcription-repair coupling factor: autoinhibition and the role of RNA polymerase. Nucleic Acids Res *35*, 1802-1811.

Thiagalingam, S., and Grossman, L. (1991). Both ATPase sites of Escherichia coli UvrA have functional roles in nucleotide excision repair. J Biol Chem *266*, 11395-11403.
